# Treatment of Infections Due to MDR Gram-Negative Bacteria

**DOI:** 10.3389/fmed.2019.00074

**Published:** 2019-04-16

**Authors:** Matteo Bassetti, Maddalena Peghin, Antonio Vena, Daniele Roberto Giacobbe

**Affiliations:** ^1^Clinica Malattie Infettive, Azienda Sanitaria Universitaria Integrata di Udine, Presidio Ospedaliero Universitario Santa Maria della Misericordia, Udine, Italy; ^2^Department of Health Sciences, University of Genoa, Genoa, Italy

**Keywords:** gram-negative, ICU, MDR, antimicrobial resistance, *Pseudomonas*, *Acinetobacter*, *Klebsiella*

## Abstract

The treatment of multidrug-resistant Gram-negative bacteria (MDR-GNB) infections in critically ill patients presents many challenges. Since an effective treatment should be administered as soon as possible, resistance to many antimicrobial classes almost invariably reduces the probability of adequate empirical coverage, with possible unfavorable consequences. In this light, readily available patient's medical history and updated information about the local microbiological epidemiology remain critical for defining the baseline risk of MDR-GNB infections and firmly guiding empirical treatment choices, with the aim of avoiding both undertreatment and overtreatment. Rapid diagnostics and efficient laboratory workflows are also of paramount importance both for anticipating diagnosis and for rapidly narrowing the antimicrobial spectrum, with de-escalation purposes and in line with antimicrobial stewardship principles. Carbapenem-resistant Enterobacteriaceae, *Pseudomonas aeruginosa*, and *Acinetobacter baumannii* are being reported with increasing frequencies worldwide, although with important variability across regions, hospitals and even single wards. In the past few years, new treatment options, such as ceftazidime/avibactam, meropenem/vaborbactam, ceftolozane/tazobactam, plazomicin, and eravacycline have become available, and others will become soon, which have provided some much-awaited resources for effectively counteracting severe infections due to these organisms. However, their optimal use should be guaranteed in the long term, for delaying as much as possible the emergence and diffusion of resistance to novel agents. Despite important progresses, pharmacokinetic/pharmacodynamic optimization of dosages and treatment duration in critically ill patients has still some areas of uncertainty requiring further study, that should take into account also resistance selection as a major endpoint. Treatment of severe MDR-GNB infections in critically ill patients in the near future will require an expert and complex clinical reasoning, of course taking into account the peculiar characteristics of the target population, but also the need for adequate empirical coverage and the more and more specific enzyme-level activity of novel antimicrobials with respect to the different resistance mechanisms of MDR-GNB.

## Introduction

In the last 15 years, intensivists, and infectious diseases consultants have started to face novel peculiar challenges in the treatment of severe infections in critically ill patients in intensive care units (ICU), due to the selection and diffusion of multidrug-resistant Gram-negative bacteria (MDR-GNB) (1, 2).

Indeed, although the development of resistance has accompanied antimicrobial therapy since its dawn, only in recent years GNB have started, in non-negligible numbers, to manifest concomitant resistance to all commonly used classes of antimicrobials. This has forced clinicians to consider treatment approaches based on combinations of drugs with impaired activity, and/or to rediscover old drugs with suboptimal pharmacokinetics and toxicity issues, all in the absence of high-level evidence to firmly guide bedside decisions (3, 4). Fortunately, this situation has started to change very recently, owing to the introduction into the market of novel drugs with potent activity against some MDR-GNB such as carbapenem-resistant *Enterobacterales* (CRE) and carbapenem-resistant *Pseudomonas aeruginosa* (CRPA) (5, 6).

Nonetheless, the availability of novel agents does not automatically imply an easy and always successful treatment, for several reasons: (*i*) most of available novel agents have still suboptimal activity against carbapenem-resistant *Acinetobacter baumannii* (CRAB); (*ii*) activity against CRE of novel β-lactam/β-lactamase inhibitors (BL-BLI) is dependent of the type of carbapenemase conferring resistance to carbapenems; (*iii*) resistance to novel antibacterials has already started to emerge, and widespread use of novel antibacterials should thus be avoided, in order to relieve selective pressure for further development of resistance; (*iv*) on the other hand, adequate coverage for MDR should be empirically guaranteed in critically-ill patients with severe infections and risk factors for MDR, in order not to delay active treatment (7–9). With so many factors at stake, treatment of MDR-GNB infections in critically-ill patients are becoming a very complex task, which requires dedicated expertise, as well as an always updated knowledge of the patients' medical history and the local microbiology epidemiology, in order to promptly recognize the risk of MDR-GNB and also the most likely resistance mechanisms involved.

This latter factor is particularly important in light of the renewed possibility of treating severe MDR-GNB infections with beta-lactams (some already available such as ceftazidime/avibactam, ceftolozane/tazobactam, and meropenem/vaborbactam, and others that will be available in the near future), that inevitably raise the question as to whether the type of suspected resistance determinant (e.g., the type of carbapenemase) should not only guide the choice of the better agent/s to be administered, but also the decisions about escalation and de-escalation, in order to follow antimicrobial stewardship purposes also at the enzyme-level.

In this review, we discuss both current and future therapeutic approaches to severe MDR-GNB infections in critically-ill patients in ICU.

## Methods

As the basis for the present narrative review, a literature search was performed in the PubMed/MEDLINE database using various combinations of pertinent keywords (e.g., “ICU,” “Gram-negative,” “therapy,” “management,” “novel antibiotics,” “novel drugs,” “*Pseudomonas*,” “*Acinetobacter*,” “*Klebsiella*,” “MDR”). Subsequently, retrieved papers were discussed and further iterative searches were conducted. Ultimately, two main narrative chapters were organized as follows: (*i*) current treatment options for MDR-GNB in critically-ill patients; (*ii*) future treatment options for MDR-GNB in critically-ill patients.

## Current Treatment Options for MDR-GNB in Critically-ill Patients

**Figure 1:**
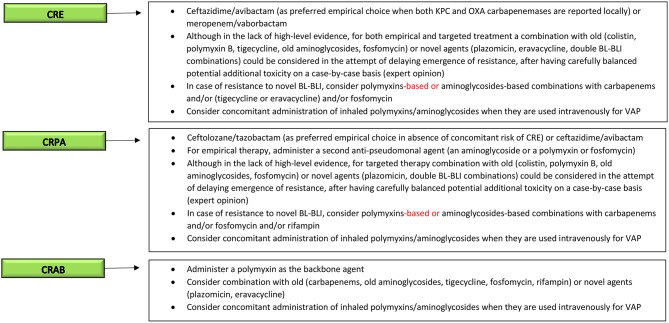
Current clinical reasoning for the treatment of serious MDR-GNB infections in critically-ill patients. MDR-GNB, Multi-drug resistant Gram-negative bacteria; CRE, carbapenem-resistant *Enterobacterales*; CRPA, carbapenem-resistant *Pseudomonas aeruginosa*; CRAB, carbapenem-resistant *Acinetobacter baumannii*; BL-BLI, β-lactam/β-lactamase inhibitors; VAP, ventilator-associated pneumonia.

The main characteristics of the currently available agents for the treatment of severe MDR-GNB infections in critically-ill patients are briefly introduced in the following paragraphs, while an example of possible clinical reasoning for guiding the treatment of MDR-GNB infections in critically-ill patients with currently available options is shown in Figure 1.

### Polymyxins

Polymyxins acts as detergents of the outer membrane of GNB, exerting bactericidal activity. Among those available for use in humans are colistin (polymyxin E) and polymyxin B. They were frequently used for the treatment of MDR-GNB infections in the past few years, when they often remained among the few (sometimes the only one) dependable options for CRE, CRPA, and CRAB (5, 10, 11). Nowadays, they are still among the first-line treatment options for CRAB infections (pending approval of more effective agents), whereas for CRE and CRPA already available novel agents should be preferred whenever possible, owing to the potential polymyxin-associated risks either of nephrotoxicity or of suboptimal concentrations (especially in the lung) (12). Furthermore, worrisome trends of increasing resistance have been reported in some countries (e.g., Italy and Greece) (13, 14). Consequently, the use of polymyxins should be optimized as much as possible in terms of dosages and indications, in order both to maximize effectiveness and to curb the emergence of further polymyxin resistance. For this reason, an international consensus document has been developed very recently, for guiding the proper use of polymyxins on all those occasions (e.g., CRAB infections, CRE and CRPA resistant to novel BL-BLI) when they still remain essential (12).

### Aminoglycosides

Aminoglycosides are concentration-dependent, bactericidal agents which inhibit the bacterial S30 ribosomal subunit. They have been frequently used in recent years for the treatment of various carbapenem-resistant GNB, frequently in case of polymyxin resistance (15, 16). However, very similar to polymyxins, two factors hamper the effective use of classical aminoglycosides (i.e., gentamicin, amikacin, tobramycin) for the treatment of MDR-GNB infections: (*i*) potential nephrotoxicity and reduced lung concentrations (17); (*ii*) increasing rates of resistance (18). With regard to this latter point, low rates of resistance have conversely been reported *in vitro* for plazomicin, a novel aminoglycoside derivative of sisomicin, which retains stability against several aminoglycoside-modifying enzymes (19). Its activity *in vitro* seems higher against CRE than against CRPA and CRAB, although possible resistance has been described in some NDM-1-producing CRE, conferred by co-expression of plazomicin-inactivating methyltransferases in these strains (5, 20). Plazomicin is currently approved by the Food and Drug Administration (FDA) for the treatment of complicated urinary tract infection (cUTI), based on the results of the phase 3 EPIC trial, in which superiority of plazomicin vs. meropenem was shown (21). In a smaller randomized study, lower mortality was observed in patients with severe CRE infections receiving plazomicin than in those receiving colistin plus tigecycline or meropenem, although the evidence was not considered substantial to achieve FDA approval for bloodstream infections (BSI) (22). An application has been recently submitted to European Medicines Agency (EMA) for approval of plazomicin for cUTI and other severe infections.

### Tigecycline

Tigecycline, a glycylcycline antibiotic which binds to the bacterial 30S ribosomal subunit, usually display activity against CRE and CRAB, but not CRPA, since *P. aeruginosa* is inherently resistant (23). Tigecycline has been used in combination with other agents for the treatment of severe CRE and CRAB infections, based mainly on favorable *in vitro* studies and observational experiences (24, 25). Of note, caution is needed in using tigecycline for ventilator-associated pneumonia (VAP), in line with an FDA warning based on pooled data from randomized clinical trials, reporting possible increased mortality in comparison with other regimens (26). When alternatives are not available and tigecycline is used for pneumonia, higher dosages should be employed for achieving sufficient PK/PD targets (27).

### Carbapenems

Although apparently paradoxical, the use of carbapenems in combinations with other agents for the treatment of carbapenem-resistant MDR-GNB was frequent in the past (before the availability of novel BL-BLI). This approach was based on the possibility of achieving sufficient carbapenem concentrations against some resistant organisms with only slightly increased carbapenem MICs, and also because of possible synergistic effects (28–31). According to the results of large observational studies, this approach seemed ultimately favorable for severe CRE infections caused by KPC-producing strains, whereas a recent randomized, controlled trial (RCT) did not find differences in survival between meropenem plus colistin vs. colistin alone for the treatment of severe CRAB infections (10, 11, 32). In this regard, the high mortality reported in the trial in our opinion further stress the need for novel effective agents against CRAB (5). There are very few small observational studies on carbapenem-including combinations for CRPA, and no firm conclusions can be drawn.

### Fosfomycin

Fosfomycin is an analog of phosphoenolpyruvate which interferes with the formation of UDP N-acetylmuramic acid, a peptidoglycan precursor. Its intravenous formulation has been used for the treatment of MDR-GNB, often in combinations with other agents, when few or none active alternatives were available (16, 33). In a small RCT including 94 patients with CRAB infections treated with colistin plus fosfomycin vs. colistin alone, higher rates of microbiological response were observed in the combination arm, but no appreciable differences were observed in survival time (34). In a small series of 48 critically-ill patients with MDR-GNB infections who received fosfomycin (mostly in combination with tigecycline or colistin) at the dosage of 8 g every 8 h for 14 days, all-cause 28-day mortality was 37.5% (35). In our opinion, because of the absence of larger studies and the reported risk of rapid selection of resistance, it might still be prudent to reserve the use of fosfomycin for selected cases (16).

### Ceftazidime/Avibactam

Ceftazidime/avibactam is a recently marketed BL-BLI combination which is active against class A (e.g., KPC) and class D (e.g., OXA) carbapenemase-producing CRE, and demonstrated activity against some CRPA isolates (36). Ceftazidime/avibactam is approved by FDA and EMA for cUTI, complicated intra-abdominal infections (cIAI), hospital-acquired pneumonia (HAP), and VAP. Furthermore, ceftazidime/avibactam received approval by EMA for infections due to GNB in adults with limited treatment options. Although efficacy of ceftazidime-avibactam in randomized clinical trials was demonstrated against ceftazidime-resistant isolates, whereas CRE were not included, its activity against the latter is supported by the favorable results of observational studies (37–39). For example, a lower 30-day mortality was observed among 104 patients with BSI due to KPC-producing *K. pneumoniae* BSI treated with ceftazidime-avibactam (within the compassionate use program) than among a matched cohort of 104 patients receiving other agents (36.5 vs. 55.7%, respectively, *p* 0.005) (39). Consequently, ceftazidime/avibactam is an important, effective, and already available option for the treatment of CRE, the use of which should be necessarily optimized according to antimicrobial stewardship principles. Indeed, some cases of resistance to ceftazidime/avibactam, conferred by *bla*_KPC_ mutations, have already been reported (8, 40).

### Meropenem/Vaborbactam

Meropenem/vaborbactam is another novel BL-BLI, exerting potent and specific activity against class A (e.g., KPC) carbapenemase-producing CRE. After having received approval by FDA for cUTI, meropenem/vaborbactam was recently approved by EMA for cUTI, cIAI, HAP, VAP, and infections due to aerobic Gram-negative organisms in adult patients with limited treatment options. In the double-blind, double-dummy TANGO-I trial, meropenem/vaborbactam demonstrated superiority vs. piperacillin/tazobactam for the treatment of cUTI, including acute pyelonephritis (41). The open-label TANGO-II trial, in which meropenem/vaborbactam was compared with best therapy available for CRE infections, was terminated early because of demonstrated superiority of meropenem/vaborbactam. Patients had mostly bacteremia, and rates of clinical cure were 65.6% in patients treated with meropenem/vaborbactam (21/32) and 33.3% in those receiving the comparators (5/15) (42). Against this backdrop, meropenem/vaborbactam is another novel and very effective option for KPC-producing CRE. Nonetheless, similarly to ceftazidime/avibactam, it should be used wisely, since resistance might develop (although possibly less frequently) (6, 9). In this light, we think future therapeutic algorithms for CRE infections should carefully take into account the peculiar characteristics and spectrum of activity of each of these two novel compounds, in order to maximize the effectiveness of anti-CRE therapies in each selected situation, as well as to preserve the activity of both drugs in the long-term.

### Ceftolozane/Tazobactam

Ceftolozane/tazobactam is probably the novel, available BL-BLI with the most potent *in vitro* activity against CRPA (although not against carbapenemase-producing strains), whereas it is not active against CRE (43). Ceftolozane/tazobactam is approved by FDA and EMA for the treatment of cIAI and cUTI on the basis of the ASPECT-cIAI and ASPECT-cUTI trials (44, 45). However, the most attractive use of ceftolozane/tazobactam at the present time is perhaps the treatment of CRPA infections, also for off-label indications. Indeed, this is confirmed by the growing amount of observational post-marketing data regarding the use of ceftolozane/tazobactam for CRPA infections, in turn reflecting the frequent lack of more active in-label alternatives (46, 47). In the future, ceftolozane/tazobactam could receive approval also for HAP and VAP, since achievement of non-inferiority vs. meropenem in the ASPECT-NP trial (NCT02070757) has been recently announced (48). Finally, the possibility of using ceftolozane/tazobactam as a carbapenem-sparing option for infections due to extended-spectrum β-lactamases (ESBL)-producing *Enterobacterales* has also been proposed, that might be useful in selected scenarios pending confirmatory clinical and economic data (43).

### Eravacycline

Eravacycline (TP-434) is a novel synthetic fluorocycline, structurally similar to tigecycline, that has been recently FDA and EMA approved for the treatment of cIAIs. Eravacycline has been synthetized to evade many resistance mechanisms observed for tetracycline. It has shown to be active against most bacteria expressing the tetracycline targeting efflux channels or containing ribosomal protection mechanisms and β-lactamases (49). As such, eravacycline has been shown to be broadly active against Gram-positive, Gram-negative, and anaerobic bacteria with the exception of *P. aeruginosa* and *Burkholderia cenopacia* (50). Importantly, it has been found to be particularly successful against CRAB, and in one *in vitro* study it was the more potent than any drug tested (51). Eravacycline is highly bioavailable after oral administration (more than 90%), has a high metabolic stability and low potential drug-drug interactions. It can also be administered intravenously (51).

Clinically, eravacycline has been studies in four phase 3 clinical trials in the setting of cIAI and cUTI with conflicting results: good performance in cIAI and suboptimal in cUTI. In the IGNITE 1 study, a randomized, double blind, non-inferiority trial, intravenous eravacycline at a dose of 1.0 mg/kg every 12 h was compared with ertapenem 1 gr every 24 h. Among patients in the intent-to-treat populations, clinical cures were 86.8% for eravacycline and 87.6% for ertapenem, showing non-inferiority of eravacycline (52). In the IGNITE 2 (NCT019783938) (53) and IGNITE 3 (NCT03032510) (54) eravacycline was compared with levofloxacin and ertapenem, respectively, for the treatment of cUTI. In both studies eravacycline did not reach non-inferiority, and issues regarding the possible poor urinary tract penetration have been raised. Another randomized, phase 3 trial (IGNITE 4) of eravacycline in comparison to meropenem for the treatment of cIAI demonstrated a favorable microbiological response of 88.9 and 100% for eravacycline against infections due to Enterobacteriaceae and *A. baumannii*, respectively (55).

In our opinion, eravacycline may offer an important option for patients with cIAI caused by MDR-GNB bacteria, including CRAB. In addition, its high oral bioavailability (>90%) allows for the switch from an intravenous to an oral formulation.

### Other Treatment Options

Variable *in vitro* synergy has been reported for rifampin in combination with other agents for the treatment of MDR-GNB infections. Possible improvements in microbiological response by adding rifampin were observed in an RCT trial in which 210 patients with *A. baumannii* infections were randomized to receive either colistin or colistin plus rifampin (56). However, similar mortality was observed in the two arms (56).

Omadacycline is an aminomethylcycline recently approved by the FDA for the treatment of community-acquired bacterial pneumonia and acute skin and skin structure infections. *In vitro* activity against some MDR-GNB has been reported (57), although clinical post-marketing experience is needed to clarify as to whether this drug will have a role in future treatment algorithms for MDR-GNB.

## Future Treatment Options for MDR-GNB in Critically-ill Patients

**Figure 2:**
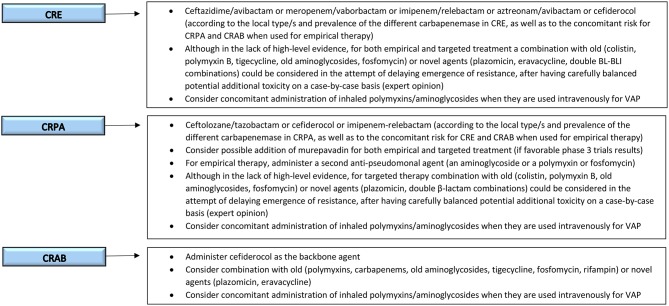
Possible future clinical reasoning for the treatment of serious MDR-GNB infections in critically-ill patients. MDR-GNB, Multi-drug resistant Gram-negative bacteria; CRE, carbapenem-resistant *Enterobacterales*; CRPA, carbapenem-resistant *Pseudomonas aeruginosa*; CRAB, carbapenem-resistant *Acinetobacter baumannii*; VAP, ventilator-associated pneumonia.

In this section we will discuss newly antibiotics against MDR Gram-negative pathogens in late stage of development. An example of possible future clinical reasoning for guiding the choice of antimicrobials to treat MDR-GNB infections in critically-ill patients, also including agents currently in phase 3, is shown in Figure 2.

### Cefiderocol

Cefiderocol is a novel siderophore antibiotic with a catechol moiety on the third position side chain which chelates free iron and provides to the new drug a unique mechanism of action (58). The catechol side chain binds to ferric iron and is actively transported across the outer membrane via the bacterial iron transporters, which are up-regulated during host immune response to acute infection (58). In addition, cefiderocol is highly active against all classes of carbapenemase (59). This combination of efficient cell entry and stability to carbapenemases allows cefiderocol to be highly active against a variety of multidrug or extremely drug resistant gram-negative bacteria, including KPC and VIM producing Enterobacteriaceae, *P. aeruginosa* producing MBL, *Stenotrophomonas maltophilia, and A. baumannii* producing OXA-type β-lactamase (59).

In a multicenter, double-blind phase 2 trial concerning treatment of cUTI, cefiderocol (2 g every 24 h) was compared with imipenem/cilastatin (1 g every 8 h) (60). Overall, 183/252 (73%) patients treated with cefiderocol and 65/119 (55%) of those treated with imipenem-cilastatin met the primary efficacy endpoint of clinical cure and microbiologic eradication at test of cure (weighted difference 18.6%, 95% CI 8.2–28.9). Moreover, cefiderocol was safe and well tolerated. Only five patients (<2%) discontinued cefiderocol because of either *C. difficile*, hypersensitivity, increased liver enzyme or diarrhea (60). Other two phase 3 clinical trials assessing the efficacy of cefiderocol for nosocomial pneumonia and serious infections caused by carbapenem- resistant gram negative pathogens are ongoing. The APEKS-NP (NCT03032380) is a randomized, double blind trial comparing all-cause mortality in adult patients with HAP/VAP due to gram-negative pathogens receiving either cefiderocol or meropenem (both in association with linezolid). The estimated date for completion of the study is June 2019. Another randomized, open label phase 3 trial (CREDIBLE-CR, NCT02714595) has been initiated in 2017 in order to provide the evidence of efficacy of cefiderocol in patients with serious infections (healthcare-associated pneumonia [HCAP], HAP, VAP, cUTI, and BSI) caused by carbapenem-resistant GNB. In this trial, cefiderocol is compared with best available therapy including up to 3 antibacterial agents for carbapenem-resistant GNB with either a polymyxin-based or non-polymyxin-based regimen.

Although definitive results from phase 3 trials are still not available, we believe that cefiderocol represents one of the most promising future therapeutic option for infections caused by serious carbapenem-resistant MDR-GNB, including CRE, CRPA, and CRAB.

### Imipenem/Relebactam

Imipenem/relebactam is a combination of an existing carbapenem (imipenem-cilastatin) with a new potent non β-lactam, bicyclic diazabicyclooctan, β-lactamase inhibitor (relebactam). Structurally related to avibactam, relebactam inhibits the activity of class A, and C beta-lactamase, but does not have activity against metallo-β-lactamases (MBL) and class D carbapenemases (61). With this profile, relebactam primarily restores the clinical activity of imipenem against imipenem-resistant isolates such as KPC- producing Enterobacteriaceae or against *P. aeruginosa* isolates showing carbapenem resistance due to porin loss in combination with AmpC expression (62, 63). However, imipenem/relebactam is not active against imipenem-resistant Enterobacteriaceae producing VIM, IMP, or NDM type of MBL as well as against *A. baumannii*, or IMP- or VIM-producing *P. aeruginosa* (62).

Relebactam' s safety, tolerability, and efficacy have been originally studied in two phase 2 clinical trials including patients with cIAI or with cUTI. In those studies, the combination of imipenem (500 mg every 6 h) with relebactam at different dosage (125 mg every 6 h and 250 mg every 6 h) was non-inferior to imipenem alone. Indeed, imipenem/relebactam showed high clinical response (>96%) in both infections and for both dosages (61, 64). In addition, the two doses (125 mg every 6 h and 250 mg every 6 h) of relebactam were well tolerated and imipenem/relebactam had the same safety profile compared with imipenem-cilastatin alone.

The RESTORE-IMI1 study was a multicenter, randomized, double-blind trial designed to assess the safety and efficacy of imipenem/relebactam compared with colistin plus imipenem for treating patients with HAP/VAP, cIAI or cUTI due to imipenem-non-susceptible bacteria (but colistin and imipenem/relebactam-susceptible). A favorable overall response for each different infection type in microbiological intent-to-treat (mMITT) population was evaluated as a primary endpoint. Preliminary results from the RESTORE-IMI1 trial have been recently presented (65). In the mMITT population, the favorable overall response was similar in the imipenem/relebactam arm (*n* = 15; 71.4%) and the colistin plus imipenem arm (*n* = 7; 70%). At day 28, imipenem/relebactam was associated with higher favorable clinical response (71.4 vs. 40%) and lower all-cause mortality (9.5 vs. 30%) compared with colistin plus imipenem. Moreover, in a safety analysis, fewer patients who received imipenem/relebactam had a drug-related adverse event (16.1 vs. 31.3%, *p* = 0.001), including treatment-emergent nephrotoxicity (10 vs. 56%, *p* = 0.001). A second pivotal phase 3 trial (RESTORE-IMI2) comparing the efficacy of imipenem/relebactam with piperacillin/tazobactam for the treatment of HAP and VAP is currently ongoing (NCT02493764).

### Murepavadin

Murepavadin (POL7080) is a protein epitope mimetic that belongs to a new class of antibiotics called outer membrane protein targeting antibiotics (OMPTA). It possesses a novel, non-lytic mechanism of action that targets the lipopolysaccharide transport protein D (LptD), involved in lipopolysaccharide biogenesis of the outer membrane of *P. aeruginosa* (66). Therefore, murepavadin is highly active against *P. aeruginosa* (66) with an expected limited impact on normal gastrointestinal flora or resistance selection in other bacterial pathogens. *In vitro*, it has demonstrated potent antimicrobial activity against *P. aeruginosa*, including carbapenemase-producing, colistin-resistant, extremely drug-resistant and pan drug-resistant isolates (67, 68) and other tested *Pseudomonas* species, but is not active against other non-fermenting Gram-negative pathogens or Enterobacteriaceae (69). The drug has a high volume of tissue distribution with linear and dose-proportional pharmacokinetics, and a half-life of 2–5 h (69).

Clinically, murepavadin has been studied in patients with VAP due to suspected or documented *P. aeruginosa*. In an open-label phase 2 study, murepavadin plus standard of care was administered in 25 patients with suspected of confirmed *P. aeruginosa* VAP (NCT02096328). Among 12 patients with confirmed *P. aeruginosa* VAP (nine of which the infection was caused by a MDR or extremely drug-resistant isolate), clinical cure was achieved in 91% of the patients at test of cure and 28-day all-cause mortality was of 8%, far below the 20–40% expected mortality rate (70). No development of treatment-emergent resistance to murepavadin was observed during the study. The PRISM-UDR trial assessing the efficacy of murepavadin with ertapenem in the treatment of nosocomial pneumonia (NCT03582007) and the PRIMS-MDR trial comparing murepavadin plus one anti-pseudomonas antibiotic with two anti-pseudomonas antibiotics for VAP are ongoing (NCT03409679).

In conclusion, if the promising results observed in phase 2 trials will be confirmed in phase 3 studies, murepavadin could represent an important option in combination with other antibiotics for empirical treatment in patients with strong risk factors for *P. aeruginosa* infections, as well as for targeted treatment. Efficacy data for other type of infection, including bloodstream infections, cUTI or cIAI, are clearly needed.

### Aztreonam/Avibactam

Aztreonam is the only available monobactam antibiotic that has been approved for treatment of gram infection since 1986. It is active against MBL-producing bacteria, but it is hydrolyzed by Ambler class A beta-lactamases (e.g., ESBL and KPCs) and class C (e.g., AmpC) beta-lactamases. Therefore, the combination of aztreonam with avibactam is able to inhibit cell wall synthesis in MBL-producing strains despite the presence of other co-carried beta-lactamases or carbapenemases (59). Aztreonam/avibactam showed a potent *in-vitro* activity against ESBL, class C b-lactamase, MBL, and KPC-producing strains with an activity 10 times that of aztreonam alone. Despite this, limited activity has been shown against *A. baumannii* or *P. aeruginosa* compared with aztreonam alone (59).

A phase III clinical trial to compare aztreonam/avibactam (with or without metronidazole) with meropenem (with or without colistin) for the treatment of HAP, VAP, and cIAI due to Gram-negative bacteria for which there are limited, or no treatment options is ongoing (REVISIT study, NCT03329092). Another phase 3 study to determine the efficacy, safety, and tolerability of aztreonam/avibactam vs. best available therapy in the treatment of serious infections (cIAI, NP, HAP, VAP, cUTI, or BSI) due to MBL–producing Gram-negative bacteria will be performed starting in April 2020 (NCT03580044).

Aztreonam/avibactam may represent an interesting alternative for infections caused by MBL-producing strains.

### Agents in Earlier Stages of Development

Agents in earlier stages of development are not the focus of the present review and have been comprehensively summarized elsewhere (5, 71). Nonetheless, it should be reminded that they could represent another precious addition to the clinicians' armamentarium in the future. For example, cefepime/zidebactam has shown promising activity against CRE, CRPA, and CRAB, and both cefepime/zidebactam and meropenem/nacubactam have demonstrated *in vitro* activity against MBL-producing CRE (5, 72–76). Ceftaroline/avibactam, cefepime/AAI101, TP-6076, VNXR-5133, WCK-5153, MEDI3902, COT-143, and RX-P2382 are other promising agents to be further evaluated for their activity against MDR-GNB during clinical development (5, 77, 78).

## Conclusions

The treatment of MDR-GNB infections in critically ill patients presents many challenges: (i) an effective treatment should be administered as soon as possible, but resistance to many antimicrobial classes invariably reduces the probability of adequate empirical coverage, with possible unfavorable consequences; (ii) there is an increasing need for rapid diagnostics and efficient laboratory workflows to anticipating diagnosis rapidly narrowing the antimicrobial spectrum, in line with antimicrobial stewardship principles; (ii) infection-control initiatives are also of paramount important, since CRE, CRPA, and CRAB are being reported with increasing frequencies worldwide, although with important variability across regions and even hospitals.

Novel treatment options have become available in recent years, providing some much-awaited resources for effectively counteracting some severe MDR-GNB infections. However, their optimal use should be guaranteed in the long term, for delaying as much as possible the emergence and diffusion of resistance to novel agents. Despite important progresses, PK/PD optimization of dosages and treatment duration in critically ill patients has still some areas of uncertainty requiring further study, that should take into account also resistance selection as a major endpoint. Treatment of severe MDR-GNB infections in critically ill patients in the near future will require an expert and complex clinical reasoning, of course taking into account the peculiar characteristics of the target population, but also the need for adequate empirical coverage and the more and more specific enzyme-level activity of novel antimicrobials with respect to the different resistance mechanisms of MDR-GNB.

## Author Contributions

All authors contributed equally to the conception and overview of the manuscript content, and also contributed equally to the writing of the manuscript. In addition, MB provided critical revision for intellectual content, and oversight. All the authors approved the final version of the manuscript.

### Conflict of Interest Statement

Outside the submitted work, MB has received funding for scientific advisory boards, travel and speaker honoraria from Angelini, AstraZeneca, Bayer, Cidara, Cubist, Pfizer, Menarini, MSD, Nabriva, Paratek, Roche, Shionogi, Tetraphase, The Medicine Company and Astellas Pharma Inc. Outside the submitted work, DRG reports honoraria from Stepstone Pharma GmbH and an unconditioned grant from MSD Italia. The remaining authors declare that the research was conducted in the absence of any commercial or financial relationships that could be construed as a potential conflict of interest.

## Abbreviations

ICU: intensive care units
MDR: multidrug-resistant
GNB: Gram-negative bacteria
CRE, carbapenem-resistant *Enterobacterales*, CRPA: carbapenem-resistant *Pseudomonas aeruginosa*
CRAB: carbapenem-resistant *Acinetobacter baumannii*
β-lactam/β-lactamase inhibitors (BL-BLI)
FDA: Food and Drug Administration
cUTI: complicated urinary tract infections
BSI: bloodstream infections
EMA: European Medicines Agency
RCT: randomized controlled trial
VAP: ventilator-associated pneumonia
cIAI: complicated intraabdominal infections
HAP: hospital-acquired pneumonia
ESBL: extended-spectrum β-lactamases
HCAP: healthcare-associated pneumonia
metallo-β-lactamases (MBL)
mMITT: microbiological intent-to-treat
OMPTA: outer membrane protein targeting antibiotics
LptD: lipopolysaccharide transport protein D
NP: nosocomial pneumonia.
